# Evaluation of the immune response induced by a mite derived fusion protein in BALB/c mice

**DOI:** 10.1186/1939-4551-8-S1-A18

**Published:** 2015-04-08

**Authors:** Dalgys Martinez, Leonardo Puerta, Ines Benedetti, Marlon Munera, Luis Caraballo

**Affiliations:** 1 Institute for Immunological Research, University of Cartagena, Colombia; 2 Foundation for the Development of Medical and Biological Sciences, Colombia; 3 University of Cartagena, School of Medicine, Colombia

## Background

The mite *Dermatophagoides pteronyssinus* is an important source of allergens and a major risk factor for allergic rhinitis and asthma. A more effective and safe way for allergen specific immunotherapy could be using recombinant allergens or their derivatives, such as hybrid molecules. This study was aimed to evaluate the immune response induced in mice by a hybrid recombinant protein, harboring several segments of major allergens of *D. pteronyssinus*.

## Methods

We engineered a fusion protein called DPx4 assembling segments of four allergens of *D. pteronyssinus* in a single molecule. Three 8 female BALB/c mice groups were immunized on days 0, 7, 14 and 21 as follows: group 1 with 10 ug of DPx4, group 2 with 20 ug *D. pteronyssinus* extract and group 3 with PBS, antigens and PBS were adsorbed to Al(OH)_3_. After immunization each animal was challenged four times intranasally. Total IgE, IgG2a and IgG1 levels were determined by ELISA. Bronchial hyperreactivity was evaluated by methacholine challenge and measurement of means enhanced pause (Penh). Mucus production in lung was evaluated with periodic acid-Schiff (PAS) and cellular infiltrate by hematoxylin-eosin staining.

## Results

Animals treated with DPx4 showed significantly lower total IgE and IgG 1 levels compared with mice treated with extract, while the IgG2a levels were higher but not statistically significant. Administration of DPx4 induced lower, but not statistically significant, bronchial hyperreactivity than that induced by administration of *D. pteronyssinus*. Treatment with DPx4 induced significantly lower inflammation around the peribronchial and perivascular zones than treatment with extract. The PAS staining revealed goblet cell hyperplasia and mucus hypersecretion in the bronchi of mice treated with *D. pteronyssinus* extract but not in mice treated with DPx4**.**

## Conclusions

The administration of DPx4 in a mice model of asthma induced a less bronchial inflammatory immune response than the mite extract, suggesting that it has potential value for the development of a mite allergen vaccine. Funded by Colciencias Grant No. 385-2009.

